# Gene–Dose Effect of *MEFV* Gain-of-Function Mutations Determines *ex vivo* Neutrophil Activation in Familial Mediterranean Fever

**DOI:** 10.3389/fimmu.2020.00716

**Published:** 2020-06-11

**Authors:** Iris Stoler, Judith Freytag, Banu Orak, Nadine Unterwalder, Stephan Henning, Katrin Heim, Horst von Bernuth, Renate Krüger, Stefan Winkler, Patience Eschenhagen, Eva Seipelt, Marcus A. Mall, Dirk Foell, Christoph Kessel, Helmut Wittkowski, Tilmann Kallinich

**Affiliations:** ^1^Department of Pediatric Pulmonology, Immunology and Critical Care Medicine, Charité – Universitätsmedizin Berlin, Berlin, Germany; ^2^SPZ (Center for Chronically Sick Children), Charité – Universitätsmedizin Berlin, Berlin, Germany; ^3^LaborBerlin – Charité Vivantes GmbH, Immunology Department, Berlin, Germany; ^4^Pediatric Gastroenterology, Nephrology and Metabolic Diseases, Charité – Universitätsmedizin Berlin, Berlin, Germany; ^5^Department of Infectious Diseases and Respiratory Medicine at Charité – Universitätsmedizin Berlin, Berlin, Germany; ^6^Department of Pediatrics, University Hospital Carl Gustav Carus, Technische Universität Dresden, Dresden, Germany; ^7^Immanuel Krankenhaus Berlin, Rheumatologie, Berlin, Germany; ^8^Department of Pediatric Rheumatology and Immunology, University Hospital Münster, Münster, Germany; ^9^Deutsches Rheuma-Forschungszentrum Berlin, A Leibniz Institute (DRFZ), Berlin, Germany

**Keywords:** autoinflammation, familial mediterranean fever, Neutrophil, S100A12, IL-18

## Abstract

Familial Mediterranean fever (FMF) is caused by mutations within the Mediterranean fever (*MEFV*) gene. Disease severity depends on genotype and gene dose with most serious clinical courses observed in patients with M694V homozygosity. Neutrophils are thought to play an important role in the initiation and perpetuation of inflammatory processes in FMF, but little is known about the specific characteristics of these cells in FMF patients. To further characterize neutrophilic inflammatory responses in FMF and to delineate gene–dose effects on a cellular level, we analyzed cytokine production and activation levels of isolated neutrophils derived from patients and subjects with distinct *MEFV* genotypes, as well as healthy and disease controls. Serum levels of interleukin-18 (IL-18) (median 11,485 pg/ml), S100A12 (median 9,726 ng/ml), and caspase-1 (median 394 pg/ml) were significantly increased in patients with homozygous M694V mutations. Spontaneous release of S100A12, caspase-1, proteinase 3, and myeloperoxidase (MPO) was restricted to *ex vivo* cultured neutrophils derived from patients with two pathogenic *MEFV* mutations. IL-18 secretion was highest in patients with two mutations but also increased in neutrophils from healthy heterozygous MEFV mutation carriers, exhibiting an *ex vivo* gene–dose effect, which was formerly described by us in patients' serum. CD62L (l-selectin) was spontaneously shed from the surface of *ex vivo* cultured neutrophils [median of geometric mean fluorescence intensity (gMFI) after 5 h: 28.8% of the initial level]. While neutrophils derived from healthy heterozygous mutation carriers again showed a gene–dose effect (median gMFI: 67.1%), healthy and disease controls had significant lower shedding rates (median gMFI: 83.6 and 82.9%, respectively). Co-culture with colchicine and/or stimulation with adenosine triphosphate (ATP) and lipopolysaccharide (LPS) led to a significant increase in receptor shedding. Neutrophils were not prevented from spontaneous shedding by blocking IL-1 or the NLRP3 inflammasome. In summary, the data demonstrate that *ex vivo* cultured neutrophils derived from FMF patients display a unique phenotype with spontaneous release of high amounts of IL-18, S100A12, MPO, caspase-1, and proteinase 3 and spontaneous activation as demonstrated by the loss of CD62L. Neutrophilic activation seems to be independent from IL-1 activation and displays a gene–dose effect that may be responsible for genotype-dependent phenotypes.

## Introduction

The prototypic autoinflammatory disease familial Mediterranean fever (FMF) is caused by pyrin-encoding *MEFV* (Mediterranean fever) gene mutations (1, 2). Recurrent self-limiting acute flares of inflammatory disease with involvement of serosal membranes and fever are key characteristics of FMF (3). Within FMF, there is wide clinical and genetic heterogeneity, but the most common mutation Met694Val (M694V) is associated with the most severe clinical phenotype in a homozygous state. FMF patients homozygous for M694V present with more joint and skin involvement, higher acute phase reactants during a clinically inactive disease, a higher rate of secondary amyloidosis, and a higher colchicine dose requirement compared to patients with other genotypes (4–6). Neutrophils are the main tissue-infiltrating cells during FMF attacks and therefore the most likely responsible for a large proportion of the observed inflammatory symptoms (7). RNA analysis of isolated short-time cultured neutrophils from patients with FMF revealed an altered spontaneous gene expression profile, for example, caspase-1, c-FOS, TLR2, and MMP9, when compared to control neutrophils (8).

Self-activation of the pyrin inflammasome and subsequent enhanced maturation of interleukin-1β (IL-1β) is of central importance in the pathophysiology of FMF (9). Elevated IL-1β secretion has been described from monocytes and neutrophils derived from FMF patients and has been identified as the main cytokine driving disease pathology in an FMF mouse model (9–11). During inflammatory attacks, neutrophils from FMF patients release neutrophil extracellular traps (NET) containing IL-1β (12, 13). Nevertheless, measuring IL-1β levels in serum from patients is hardly possible (14), and a constitutive pyrin inflammasome activation in patient macrophages *ex vivo* has not been described (15).

In a previous study, we reported unstimulated neutrophils from homozygous M694V patients to spontaneously release higher levels of the IL-1 family cytokine IL-18, caspase-1, and myeloid cell-derived S100A12 compared to neutrophils from healthy controls (HCs) *in vitro* (11). In addition, highly elevated serum levels of these proteins can be detected in the serum of FMF patients and were shown to differentiate clinical status and genotype (11).

These results raised the hypothesis that neutrophils carrying *MEFV* mutations do exhibit a highly characteristic activation status. In order to further decipher the neutrophilic inflammatory response in FMF and to delineate the gene–dose effect of *MEFV* mutations, we analyzed the spontaneous and induced cytokine secretion by neutrophils derived from patients and controls. Furthermore, the activation state of neutrophils was determined by measuring the density of surface molecules. With these analyses, we addressed the following objectives: (i) the spontaneous marker release and change of surface marker expression are restricted to neutrophils derived from patients with FMF, (ii) the amount of spontaneous neutrophilic activation depends on a gene–dose effect, and (iii) the spontaneous release of inflammatory markers is restricted to a specific set of proteins. For these reasons, we included the analysis of neutrophils derived from patients with other chronic active inflammatory disorders, for example, Crohn's disease, rheumatic diseases, cystic fibrosis, autoinflammatory diseases, and immunodeficiencies with chronic inflammation, as well as acute infections.

## Patients and Methods

### Patients and Control Groups

HCs (*n* = 9, mean age 42 years), healthy heterozygous *MEFV* carriers (*n* = 6, mean age 45 years), and patients with FMF and two pathogenic mutations (*n* = 12, mean age 19 years) or other diverse inflammatory diseases [infections *n* = 6, cystic fibrosis *n* = 5, Crohn's disease *n* = 4, rheumatic diseases *n* = 4, tumor necrosis factor receptor-associated periodic syndrome (TRAPS) *n* = 2, and immunodeficiencies with chronic inflammation *n* = 2, mean age of all 41 years] were recruited at the Children's Hospital and the Clinic for Pneumology and Infectious Diseases (both Charité Berlin) as well as the Immanuel Hospital Berlin Buch. For patients' characteristics, see Table 1, Table S1. Clinical status was assessed by a standardized questionnaire. In patients with chronic inflammatory diseases other than cystic fibrosis, infections within the last 2 weeks prior to blood sampling were excluded. In Crohn's disease, disease activity was assessed by use of the Harvey–Bradshaw index, which captures general well-being, abdominal pain, number of liquid stools per day, abdominal mass, and complications (mild 5–7, moderate 8–16, and severe >16) (17). In patients with cystic fibrosis, severity of pulmonary exacerbation was assessed by changes in (1) sputum volume or color, (2) cough, (3) malaise and/or fatigue, (4) weight loss, (5) decrease in FEV_1_ ≥10% or radiographic changes, and (6) dyspnea (maximal count 6) (18). In patients with rheumatoid arthritis, disease activity was measured by disease activity score 28 (DAS 28), which summarizes (1) number of tender joints (0–28), (2) number of swollen joints (0–28), (3) C-reactive protein (CRP) (mg/L), and (4) subjective evaluation of disease activity by the patient (0–100 visual analog scale). Patients who received >5 mg/day prednisolone equivalent were excluded.

**Table 1:** Characteristics of the patients in the core study cohort.

	**Controls**	**Asymptomatic heterozygous M694V carriers**	**Patients with two mutations within *MEFV* other than M694V homozygosity**	**Homozygous M694V FMF patients**	**Other inflammatory diseases (total)**	**Infections**	**Cystic fibrosis**	**Crohn's disease**	**Rheumatic diseases**	**TRAPS**	**Immunodeficiencies**
Patients, no	9	6	7	5	23	6	5	4	4	2	2
No. male/no. female	4/5	3/3	4/3	4 /1	19/4	6/0	4/1	4/0	2/2	1/1	2/0
Age at inclusion, mean (range) years	42 (27–61)	45 (40–51)	19 (15–29)	19 (15–25)	41 (10–82)	59 (37–82)	32 (19–47)	23 (16–41)	54 (19–74)	26 (10–41)	41 (24–53)
Mean severity (SD)	n.a.	n.a.	Attack frequency/in last 12 months: 0	Attack frequency/in last 12 months: 3 (6)	n.a.	n.a.	Bilton score: 4 (0.71)	Harvey–bradshaw score: 6.75 (1.50)	DAS 28: 4.3 (0.50)	n.a.	n.a.
Genotype	n.a.	6 × heterozygous M694V	2 × M694V/V726A, 2 × M694V/M680I, 1 × M694V/V726A/E148Q, 1 × M694V/A744S, 1 × M680I homozygous	M694V homozygous	n.a.	n.a.	1 × F508del and 1717-1 G>A, 4 × F508del/ F508del	n.a.	n.a.	2 × heterozygous T50M	1 × gp91phox
**Inflammation markers in serum**											
CRP, median (range), mg/L	1.0 (<0.3–6.0)	3 (0.8–5.8)	3 (0.3–8)	82.1 (1.3–112.5)	18.4 (0.3–182.8)	84.9 (39.6–182.8)	18.4 (8.2–66.1)	14.9 (2.9–55.5)	9.5 (3.0–50.7)	1.0 (0.3–1.9)	5.2 (1.5–8.9)
S100A12, median (range), ng/ml	362 (55–966)	456 (236–570)	710 (354.6–43,154)	9,725 (1,267–12,864)	325 (73–1,189), *n* = 8	*n* = 0	568 (375–761), *n* = 2	236, *n* = 1	471 (276–1,189), *n* = 3	95 (73–116)	*n* = 0
Caspase-1, median (range), pg/ml	158 (53–254)	263 (140–275)	207 (107–424)	394 (265–1,552)	157 (59–108)	154 (75–800)	157 (103–302)	119 (64–257)	251 (165–31)	62 (59–64)	113 (65–161)
IL-18, median (range), pg/ml	189 (106–243)	442 (245–651)	2,699 (571–7,322)	11,485 (4,054–18,028)	309 (138–15,221)	397 (139–1,350)	261 (138–398)	209 (192–493)	5,163 (758–15,221)	338 (306–370)	381 (227–535)

*CRP, C-reactive protein; IL-18, interleukin-18*.

*Cutoff for inflammation markers: CRP <5 mg/l; S100A12 <140 ng/mg. IL-18 in healthy controls is 169 pg/ml [117, 243] (16). In case of incomplete data on individual serum marker concentration, the number of analyzed samples is indicated*.

### Ethical Approval

This study was approved by the ethical commission of the Charité – Universitätsmedizin Berlin (Ref: EA2/033/09). Written informed consent was obtained from all HCs, patients, and/or their parents or legal guardians.

### *In vitro* Analyses

Neutrophils were isolated by a two-density centrifugation using Percoll (GE Healthcare, Freiburg, Germany) within 30–60 min after blood drawing (for the experimental approach, see Figure S1). Cells were counted, and purity was determined by cell-counting flow cytometry (Sysmex). An additional serum sample was aliquoted, immediately frozen, and stored at −80°C for later analysis of inflammatory mediators. Neutrophils (5 × 10^6^ cells/ml) were left untreated or stimulated for 5 h with phorbol myristate acetate (PMA) (10 nM; Sigma-Aldrich, Munich, Germany) or lipopolysaccharide (LPS) (10 ng/ml LPS-RS Ultrapure; InvivoGen), with or without the addition of colchicine (5 μg/ml; Sigma) at time 0 and with or without the addition of adenosine triphosphate (ATP) disodium salt (Sigma-Aldrich) at 3.5 h. Cells were harvested after 30 min and 1, 2, 3, 4, or 5 h, respectively. In HC, heterozygous *MEFV* mutation carriers and FMF patients cell viability were determined by microscopy after Trypan blue staining and flow cytometry after propidium iodide and annexin staining (Becton-Dickinson. Heidelberg, Germany).

Expressions of S100A12, IL-18, and IL-1β were analyzed in neutrophils derived from a previously described cohort of HCs and FMF patients with active disease (numbering in Table S1: controls 1.10–1.13, patients 2.14–2.19, data shown in Figure S4) (11).

After stimulation, RNA was isolated from 5 × 10^6^ neutrophils according to the user manual [“Total RNA Isolation” (Macherey-Nagel) and reverse transcribed into cDNA “RevertAid H minus First Strand cDNA Synthesis Kit” (Fermentas)]. RT-PCR was performed with the ABI PRISM 7900HT Sequence Detection System after adding primers, SYBR FAST qPCR and SYBR Green by KAPA Biosystems.

Measurement cycle threshold (Ct) in comparison to housekeeping genes glyceraldehyde 3-phosphate dehydrogenase (GAPDH) und ribosomal protein L (RPL) was analyzed (ΔCt). The following primers were used: GAPDH 236 forward 5′-GCA AAT TCC ATG GCA CCG T-3′, GAPDH 339 reverse 5′-GCC CCA CTT GAT TTT GGA GG-3′, RPL 13A 277 forward 5′-AGG TAT GCT GCC CCA CAA AAC-3′, RPL 13A 418 reverse 5′-TGT AGG CTT CAG ACG CAC GAC-3′, IL-1β forward: 5′-GCG GCC AGG ATA TAA CTG ACT TC-3′, IL-1β reverse 5′-TCC ACA TTC AGC ACA GGA CTC TC-3′, IL-18 forward 5′-TTC AAC TCT CTC CTG TGA GAA CA-3′, IL-18 reverse 5′-ATG TCC TGG GAC ACT TCT CTG-3′, S100A12 reverse 5′-TGT TTG CAA GCT CCT TTG TAA GC-3′, and S100A12 73 forward 5′-CAA AAC TTG AAG AGC ATC TGG AGG-3′.

### Analysis of Inflammatory Mediators

ELISAs following the manufacturers' standard protocols were performed in patients' and control serum for S100A12 (Circulex, Nagano, Japan), IL-18 (human IL-18 ELISA kit, MBL, Woburn, USA), and caspase-1 (Human Caspase-1/ICE Immunoassay, R&D, Abingdon, UK).

Cytokines or cytokine receptor antagonists (IL-1β, IL-6, IL-8, IL-10, IL-18, IL-1Ra, and TNFα), neutrophilic granula proteins (Proteinase 3, MPO), and chemokines [MCP-1, MIP-1α (CCL-3), MIP-1β (CCL-4), and MIP-3α (CCL-20)] in culture supernatants (SNs) were quantified by multiplexed bead array assays (ProcartaPlex, Thermo Fisher, Waltham, MA, USA; R&D Systems, Minneapolis, MN, USA) according to the manufacturers' instructions. S100A12 was detected by a combination of in-house monoclonal anti-S100A12 antibodies (19) translated to the MagPlex microsphere platform (Luminex, Hertogenbosch, The Netherlands) (20). Data acquisition was performed on a MagPix instrument (Merck Millipore) using xPONENT v4.2 software (Luminex). Data were analyzed using ProcartaPlex Analyst software (v1.0; eBioscience).

### Flow Cytometry

Fluorescence-activated cell sorting (FACS) analysis (Canto, FACS Diva software) was performed by the use of CD45-PE-Cy5, CD11b-APC, CD16-PC7, and CD62L-FITC antibodies and isotype staining by use of mIgG1-PE and mIgG1-APC. mIgG1-FITC and CD62L-FITC were purchased from Becton Dickinson; all other antibodies were purchased from Beckman Coulter. In the neutrophil-enriched cell population, granulocytes were positively distinguished from cell debris, and lymphocytes by positive staining with CD45 and high side scatter. Eosinophilic granulocytes were differentiated by the expression of CD16. To identify activation, neutrophils were stained with CD11b and CD62L (Figure S2). The gate for the isotype control was set to exclude 99% of the total population.

### Statistical Analysis

Data were analyzed with GraphPad Prism software (Version 8.0 for Mac OS X, GraphPad Software, La Jolla, CA, USA), and tests applied as indicated in figure legends. Significance of differences in serum levels of inflammatory mediators were analyzed by Kruskal–Wallis followed by Dunn's multi-comparison test. ^*^p < 0.05, ^**^p < 0.01, ^****^p < 0.0001, and *p* ≤ 0.05 were considered statistically significant.

## Results

### Serum Levels of S100A12, IL-18, and Caspase-1 Detect Inflammation in FMF

Although most of the FMF patients investigated in this cohort were well-controlled by continuous colchicine therapy (Table S1), homozygous M694V mutation carriers showed a significant increase of CRP [median 82.1 mg/L (range 1.3–112.5 mg/L), *p* < 0.05] compared to patients with other mutations or HCs (Table 1, Table S1). It was shown previously that IL-18 and S100A12 are especially sensitive to detect subclinical inflammation in patients with FMF (11, 21). In this independent cohort, serum levels of IL-18, S100A12, and, interestingly, caspase-1, a marker for inflammasome activation, were also significantly increased in patients with homozygous M694V mutations compared to controls [IL-18: median 11,485 pg/ml (4,054–18,028), *p* < 0.0001, S100A12: median 9,726 ng/ml (1,267–12,864), *p* < 0.01; caspase-1: median 394 pg/ml (265–1,552), *p* < 0.01]. Furthermore, IL-18 levels were significantly increased in patients with two mutations other than M694V homozygosity compared to healthy subjects [median 2,699 pg/ml (571–7,322), *p* < 0.01].

These observations confirmed that IL-18 and S100A12 as well as caspase-1 are increased in FMF and prompted us to analyze their secretion pattern in *ex vivo* isolated neutrophils in more detail.

### FMF Neutrophils Spontaneously Release S100A12, IL-18, and Caspase-1

Therefore, we extended our analyses in this patient cohort to *ex vivo* studies of neutrophils as a prominent source of IL-18, caspase-1, and S100A12 secretion (11). For this reason, we determined the kinetics of spontaneous protein secretion and compared findings to neutrophils derived from healthy heterozygous *MEFV* mutation carriers, as well as patients with active infections and other inflammatory diseases.

After cell preparation, neutrophils were enriched to a mean of 90.6% (SD 9.3%). The amount of monocytes, a potential contaminating source of proinflammatory cytokines, ranged at a mean percentage of 0.8% (SD 1.3%). Between the different diseases, no differences in cell distribution was observed (Table S2). The addition of ATP, LPS, or colchicine did not decrease cell viability as measured by Trypan blue staining as well as by flow cytometry after propidium bromide and annexin staining (Tables S3, S4).

**Figure 1:**
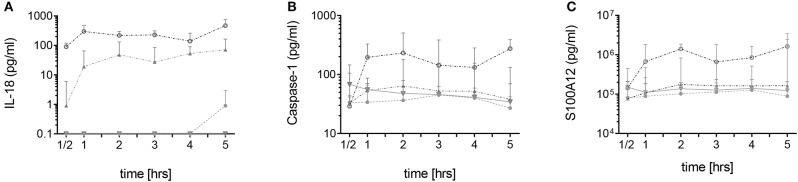
Spontaneous secretion of IL-18, caspase-1, and S100A12 by neutrophils derived from *MEFV* mutation carriers and controls. Isolated neutrophils from healthy controls (filled circles, *n* = 5–7), healthy heterozygous *MEFV* mutation carriers (filled triangles, *n* = 6), FMF patients with compound heterozygous or homozygous mutations (open circles, *n* = 9), and patients with other chronic inflammatory and infectious diseases (open triangles, *n* = 7–22) were cultured without stimulation. Secretion of IL-18 **(A)**, caspase-1 **(B)**, and S100A12 **(C)** was assessed by ELISA in the supernatants at indicated time points. Note that values for S100A12 are represented on a logarithmic scale and in pg/ml. Values are given as median and interquartile ranges.

**Figure 2:**
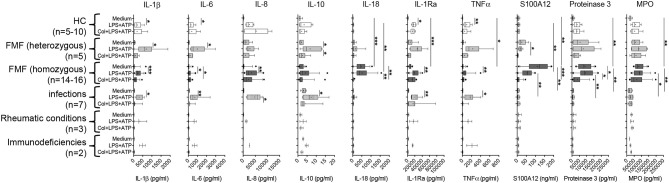
Spontaneous and induced secretion of various mediators by neutrophils derived from *MEFV* mutation carriers and patients with other inflammatory diseases. Isolated neutrophils derived from controls and from patients with indicated diseases were cultured without stimulation or were stimulated with 10 ng/ml of lipopolysaccharide (LPS) for 5 h and 1 mM adenosine triphosphate (ATP) for 90 min. Part of the cells was cultured with 5 μg/ml colchicine added at time 0. Concentrations of interleukin (IL)-1β, IL-6, IL-8, IL-10, IL-18, IL-1RA, TNFα, S100A12, proteinase 3, and MPO were quantified by multiplexed bead array assay (D). Data were analyzed by Kruskal–Wallis followed by Dunn's multi-comparison test (*p < 0.05, **p < 0.01, ***p < 0.001). Note that different assay systems were used in Figure 1 compared to Figure 2 explaining potential differences in concentrations.

In neutrophils derived from FMF patients, IL-18, caspase-1, and S100A12 were rapidly secreted during the first 60 min of culture. For IL-18, a gene/dose-dependent secretion was observed with the highest levels secreted by cells derived from patients with two pathogenic *MEFV* mutations followed by secretory activity of cells derived from healthy heterozygous carriers (Figures 1A–C). As previously described, no spontaneous increased IL-1β secretion was observed (Figure 2, Figure S3B). In neutrophils derived from patients with acute infections and other active inflammatory diseases, secretion of these inflammatory markers did not differ from HCs (Figure 2, Figure S3B).

As previously demonstrated by single ELISA (11) and confirmed in this cohort, the spontaneous secretion of S100A12 and IL-18 by neutrophils from FMF patients cannot be further enhanced by *in vitro* cell stimulation but can be reduced by the addition of colchicine (Figure 2, Figure S3B). To elucidate whether this spontaneous release is specific for these particular mediators and for neutrophils from FMF patients, we analyzed the secretion of various cytokines, chemokines, and granular proteins in neutrophils derived from patients with different inflammatory conditions (Figure 2, Figures S3A,B). IL-18, S100A12, proteinase-3, and MPO are the only proteins that were spontaneously secreted at high levels by neutrophils from FMF patients, and further stimulation with PMA did not increase protein concentration in the SN. Addition of colchicine resulted in decreased IL-18, S100A12, and proteinase-3 secretion into culture SN. In contrast, blocking the IL-1 signaling pathway or the activation of NLRP3 by the addition of Anakinra or MCC950, respectively, did not alter the secretion of these proteins (data not shown). Furthermore, no highly increased and/or specific spontaneous protein release from neutrophils derived from disease controls was observed. Additionally, unstimulated neutrophils from patients with FMF and other inflammatory diseases did not release significant concentrations of chemokines, for example, MCP-1, MIP-1α (CCL-3), MIP-1β (CCL-4), and MIP-3α (CCL-20).

**Figure 3:**
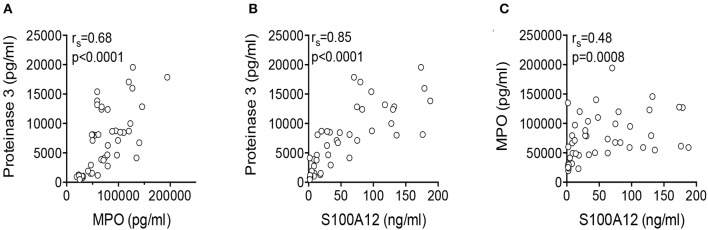
Correlation of secreted protein concentrations. Correlations of proteinase 3 and MPO **(A)**, S100A12 and proteinase 3 **(B)**, and S100A12 and MPO **(C)** in the supernatant of unstimulated neutrophils derived from FMF patients were analyzed by Spearman rank correlation.

Interestingly, spontaneous S100A12 secretion correlated with the amount of measured proteinase 3 (*r*_s_ = 0.85, *p* < 0.0001) and MPO (*r*_s_ = 0.48, *p* = 0.0008) (Figure 3).

Although we observed a marked spontaneous release of S100A12 and IL-18 from FMF neutrophils, cell stimulation only increased transcription of IL-1β but did not alter transcription levels of S100A12 and IL-18 (Figure S4).

Taken together, the spontaneous release of S100A12, IL-18, MPO, and proteinase 3 is restricted to neutrophils derived from FMF patients and, in the case of IL-18 when analyzed by ELISA, also increased in healthy mutation carriers.

### FMF Neutrophils Reveal Spontaneous Loss of Surface-Bound CD62L

**Figure 4:**
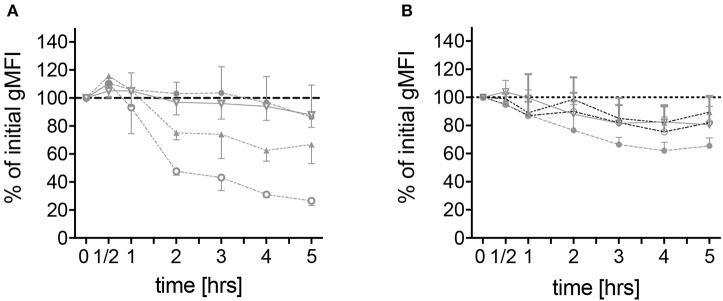
Spontaneous alteration of CD62L and CD11b expressions on neutrophils derived from *MEFV* mutation carriers and controls. Isolated neutrophils from healthy controls (filled circles, *n* = 6), healthy heterozygous *MEFV* mutation carriers (filled triangles, *n* = 6), FMF patients with compound heterozygous or homozygous mutations (open circles, *n* = 10), and patients with chronic inflammatory as well as infectious diseases (open triangles, *n* = 24) were cultured without stimulation. Geometric mean fluorescence intensity of CD62L **(A)** and CD11b **(B)** expression was measured at the indicated time points by flow cytometry. Values are given as mean and interquartile ranges.

To further characterize spontaneous neutrophilic activation, expression of surface markers on neutrophils derived from the different patient groups was measured by means of flow cytometry. CD62L, a molecule responsible for endothelial attachment and transmigration into affected tissues indicating cell activation if shed from the cell surface, was rapidly shed from the surface of neutrophils derived from FMF patients during the first 2 h of culture and to a lesser extent during the following 3 h. After 5 h, the median of the geometric mean fluorescence intensity (gMFI) reached 28.8% (range 11.4–65.4) of the initial value (Figure 4A). No difference in the level of CD62L shedding was observed when comparing FMF patients with increased CRP to those with CRP values within the normal range (data not shown). In neutrophils derived from healthy mutation carriers, CD62L expression declined more constantly over time, reaching a final median gMFI of 67.1% (range 45.7–94.6). Neutrophils from patients with infections and other active inflammatory diseases and HCs exhibited only slight CD62L shedding [median gMFI 82.9% (range 73.9–112.6) and 83.6% (61–136.0), respectively; Figure 4A, Figure S5].

Of note, one heterozygous FMF patient with persisting symptoms (one to two attacks per month despite regular colchicine intake, patient 2.13 in Table S1) demonstrated a rapid shedding of CD62L comparable to the effects observed in homozygous FMF patients and different from heterozygous healthy mutation carriers (gMFI 18.5%), indicating a possible link to disease activity beyond genotype effects.

**Figure 5:**
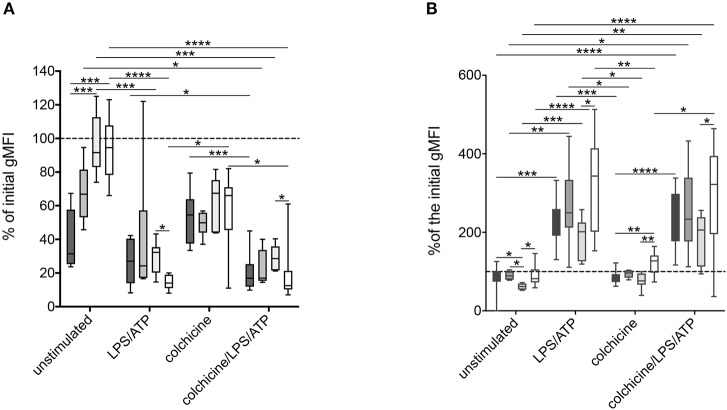
Alteration of surface markers after stimulation of neutrophils derived from *MEFV* mutation carriers and patients with other inflammatory diseases. Isolated neutrophils from FMF patients (*n* = 10, dark gray), heterozygous mutation carriers (*n* = 6, middle dark gray), controls (*n* = 7, light dark gray), and patients with other inflammatory diseases (*n* = 11, white) were cultured as described in Figure 2. After 5 h, CD62L **(A)** and CD11b **(B)** expressions were analyzed. Box-and-whisker plots depict 5th−95th percentiles. Significance was analyzed by Kruskal–Wallis followed by Dunn's multi-comparison test, *p < 0.05, **p < 0.01, ***p < 0.001, ****p < 0.0001.

Co-incubation with colchicine and/or stimulation with ATP and LPS led to a significant increase of CD62L shedding in all analyzed patient groups (Figure 5A). The addition of the IL-1 receptor antagonist anakinra and the NLRP3-inhibiting compound MCC950 did not alter this stimulation-dependent CD62L shedding (Figure S6).

As a member of the MAC-1 complex, CD11b plays a role in neutrophil recruitment and can be used as a marker of neutrophil activation (22). No difference in CD11b expression on unstimulated neutrophils from patients with different diseases and *MEFV* mutation carriers was observed (Figure 4B). Stimulation led to an increase of CD11b expression with no differences between the disease groups. In contrast to the expression of CD62L, the sole addition of colchicine did not alter CD11b levels (Figure 5B).

These observations demonstrate again a spontaneous gene/dose-dependent activation of *MEFV* mutated neutrophils, which does not depend on either autocrine IL-1 action or on induction of the NLRP3 inflammasome.

## Discussion

In this study, we confirm that IL-18 and S100A12 can sensitively detect inflammation in FMF. Furthermore, we affirm the spontaneous hypersecretion of these proteins together with caspase-1 by analyzing patients with well-controlled FMF and demonstrate that this secretion occurs rapidly within the first 2 h of culture. Since the effects of a spontaneous mediator release were not observed in neutrophils derived from patients with other highly active inflammatory diseases such as infections, Crohn's disease, rheumatic and autoinflammatory diseases, cystic fibrosis, and immunodeficiencies with chronic inflammation, these effects seem to be FMF specific. The effect of spontaneous mediator release was observed in both active (11) and mostly well-controlled (this cohort) colchicine-treated FMF patients. This indicates that therapeutically applied colchicine does not control the *ex vivo* analyzed spontaneous activation of neutrophils irrespective of the clinical phenotype. These observations are supported by a previous work demonstrating increased transcription of selected genes by short-time-cultured neutrophils derived from colchicine-treated FMF patients (8).

Many clinical observations in FMF indicate a genotype–phenotype correlation with most severe diseases observed in the presence of homozygosity of the highly pathogenic M694V mutation and with milder diseases in patients harboring other mutations or being heterozygous mutation carriers (6). Analysis demonstrating gene/dose-dependent IL-1β secretion by stimulated monocytes from FMF patients with different genotypes might in part explain these observations (10, 15). Furthermore, levels of neutrophil-derived proteins in patients' serum correlated with the underlying *MEFV* genotypes (11). This is in line with the clinical observation that S100A12 is particularly sensitive in detecting subclinical inflammation in healthy heterozygous mutation carriers (21). Additionally, neutrophils derived from patients with poorly controlled FMF spontaneously secrete high levels of S100A12 and IL-18 (11).

Both IL-1β and IL-18 exhibit proinflammatory effects, the latter one in most instances through the induction of interferon-γ (23). So far, mainly monocytes were used to study aberrant cytokine secretion in cells derived from FMF patients: long-term stimulation with LPS for 18 h—a condition which induces canonical (caspase-1-dependent) as well as non-canonical (caspase-4/5- and caspase-8-dependent) inflammasomes (24, 25) induced a strong IL-1β production in monocytes derived from FMF patients (10). Similarly, IL-18 and IL-1β secretion was enhanced in monocytes from patients with FMF when treated with the pyrin-specific stimulus *Clostridium difficile* toxin B (TcdB) as a second signal for a short time (15). The role of IL-1β in the pathogenesis of FMF has now clearly been demonstrated by the successful application of IL-1-blocking biological agents in FMF patients (26, 27). IL-1β and IL-18 as well as the S100A12 molecules lack a specific signal sequence and are therefore secreted by an ER/Golgi-independent pathway referred to as “unconventional secretion” (28, 29) or via gasdermin D (GASDMD)-mediated processes (30).

Caspase-1 processes the intracellularly located pro-IL1β/IL-18 into active cytokines (31). In macrophages, the inflammatory caspase-1, caspase-4, caspase-5, and caspase-11 have the capacity to induce GASDMD-dependent osmotic cell lysis, named pyroptosis, through the formation of large oligomeric membrane pores (32). In murine *MEFV*^V726A/V726A^ macrophage IL-1β release, pyroptosis, and autoinflammatory symptoms seem to depend entirely on GASDMD activity (33). In FMF, this process is solely controlled by dephosphorylation of pyrin through the inhibition of protein kinases PKN1/2 (34). Additionally, emerging evidence suggests that neutrophilic activation can also lead to caspase-1- and GASDMD-dependent IL-1β and IL-18 maturation and secretion without concomitant lytic cell death (35, 36). In the present study, spontaneous S100A12 and IL-18 secretion correlated with inflammasome activity as measured by an increased caspase-1 secretion *in vivo* and *ex vivo* without the occurrence of significant cell death after 5 h of culture with or without stimulation. This observation suggests a differentially regulated IL-18 and S100A12 hypersecretion in neutrophils from FMF patients involving the mutated pyrin inflammasome, as well as GASDMD. In contrast to studies performed in *ex vivo* stimulated murine neutrophils (35, 36), IL-1β was only marginally elevated in the SNs of neutrophils derived from FMF patients, suggesting monocytes rather than neutrophils are the major source of soluble IL-1β in patients with FMF (10). But still, neutrophils still contribute to IL-1β-mediated inflammation during acute FMF attacks by the release of NET-associated IL-1β driven by mechanisms of autophagy (12, 13).

Activation of neutrophils is a complex and not fully understood process involving many different receptors, for example, G-protein-coupled receptors, Fc receptors recognizing Ig-opsonized pathogens and immunocomplexes, adhesion receptors, cytokine receptors, and innate immune receptors recognizing damage-associated molecular patterns. Engagement of these receptors led to neutrophil migration, differential gene expression, reactive oxygen species (ROS) production, and exocytosis of intracellular granules and vesicles (37). The degranulation in neutrophils is a tightly controlled process characterized by a microtubule-dependent granule transport toward the cell surface and a subsequent fusion of the organelle membrane with the cell membrane (38). Azurophil granules contain myeloperoxidase and proteinase-3 (38), two proteins which have been found abundantly in the SN of unstimulated neutrophils from FMF patients. This observation suggests a spontaneous *MEFV*-dependent degranulation of *ex vivo* cultured neutrophils from patients with FMF. Although the concentrations of these molecules correlate strongly with those of the S100A12, it seems unlikely that they are secreted together since S100A12 does not appear to be stored in granules.

The selectin CD62L (l-selectin) and the integrin Mac1 (α_M_β_2_; CD11b/CD18) are involved in neutrophil attachment, rolling, and stable tethering on endothelial cells and thus play a crucial role in transmigration of neutrophils from the blood into affected tissues (39). A disintegrin and metalloproteinase 17 (ADAM17) is a type 1 transmembrane protein with a sheddase activity for the membrane-bound CD62L. In contrast to other ADAM family members, the activity of ADAM17 is greatly enhanced by cell activation, for example, via the recognition of PAMPs or TNFα, leading to a loss of membrane-bound CD62L within minutes (40, 41). In addition, neutrophil degranulation is associated with increased protein kinase-C-dependent integration and activation of ADAM17 into the cell surface membrane (40, 42). Thus, the spontaneous *ex vivo* loss of CD62L expression on the surface of neutrophils can be explained by their activation and/or by the exocytosis of granula. Of note, at the time of cell isolation, CD62L expression was not altered in neutrophils from patient with FMF compared to controls in the present study or in published data (43), indicating that the shedding process is greatly enhanced by the culture conditions that may mitigate *in vivo* regulation of CD62L surface expression.

Our study has several limitations: due to the elaborative cell preparation process, only a limited number of patients were analyzed; thus, differences between genotypes other than M694V homozygosity might have been missed. Furthermore, no patients during acute flare were analyzed. In order to translate the observed pathophysiological alterations as a marker for disease management, a simpler protocol, for example, the analysis of whole blood, must be established. In order to establish a mechanistic explanation for our observations further functional studies on neutrophils derived from FMF patients have to be performed.

In summary, our data indicate that the differential secretion of inflammatory mediators such as IL-18 and S100A12 by neutrophils with mutations in the *MEFV* gene plays an important role in the pathophysiological processes in FMF. Our *ex vivo* studies of neutrophils detected a highly inflammatory phenotype that depends on a gene–dose–response relationship. A more detailed knowledge about the role of neutrophils in the pathophysiology of FMF may contribute to the development of specific markers for functional characterization of *MEFV* variants, as well as therapy control, and thus improve patient management.

## Data Availability Statement

All datasets generated for this study are included in the article/Supplementary Material.

## Ethics Statement

The studies involving human participants were reviewed and approved by Ethikkommission der Charité – Universitätsmedizin Berlin Campus Charité Mitte, Charitéplatz 1, 10117 Berlin Geländeadresse: Virchowweg 10. Written informed consent to participate in this study was provided by the participants' legal guardian/next of kin.

## Author Contributions

IS, JF, BO, NU, SW, and CK performed most experiments. SH, HB, RK, PE, and ES recruited patients. MM, DF, CK, HW, and TK planned and supervised the study and wrote the manuscript. All authors approved the final version.

## Conflict of Interest

DF has received honoraria from Novartis, Chugai-Roche and SOBI, and he has received research funding from Novartis, Pfizer and SOBI. TK received honoraria from Roche and SOBI. HW received speaker honoraria from Novartis, Shire/Takeda, and CSL-Behring. The remaining authors declare that the research was conducted in the absence of any commercial or financial relationships that could be construed as a potential conflict of interest.

## References

[1] Ancient missense mutations in a new member of the RoRet gene family are likely to cause familial Mediterranean fever The International FMF Consortium. Cell. (1997) 90:797–807. 10.1016/S0092-8674(00)80539-5 9288758

[2] French FMFC Bernot A Clepet C Dasilva C Devaud C Petit JL Caloustian C. A candidate gene for familial mediterranean fever. Nat Genet. (1997) 17:25–31. 10.1038/ng0997-25 9288094

[3] Sohar E Gafni J Pras M Heller H. Familial Mediterranean fever. a survey of 470 cases and review of the literature. Am J Med. (1967) 43:227–53. 10.1016/0002-9343(67)90167-2 5340644

[4] Tunca M Ozdogan H Kasapcopur O Yalcinkaya F Tutar E Topaloglu R. Familial Mediterranean fever (FMF) in Turkey: results of a nationwide multicenter study. Medicine. (2005) 84:1–11. 10.1097/01.md.0000152370.84628.0c 15643295

[5] Majeed HA El-Shanti H Al-Khateeb MS Rabaiha ZA. Genotype/phenotype correlations in Arab patients with familial Mediterranean fever. Semin Arthritis Rheum. (2002) 31:371–6. 10.1053/sarh.2002.32551 12077709

[6] Federici S Calcagno G Finetti M Gallizzi R Meini A Vitale A. Clinical impact of MEFV mutations in children with periodic fever in a prevalent western European Caucasian population. Ann Rheum Dis. (2012) 71:1961–5. 10.1136/annrheumdis-2011-200977 22580583

[7] Samuels J Aksentijevich I Torosyan Y Centola M Deng Z Sood R Familial Mediterranean fever at the millennium. Clinical spectrum, ancient mutations, and a survey of 100 American referrals to the National Institutes of Health. Medicine. (1998) 77:268–97. 10.1097/00005792-199807000-00005 9715731

[8] Manukyan G Petrek M Tomankova T Martirosyan A Tatyan M Navratilova Z. Colchicine modulates expression of pro-inflammatory genes in neutrophils from patients with familial Mediterranean fever and healthy subjects. J Biol Regul Homeost Agents. (2013) 27:329–36. 23830384

[9] Chae JJ Cho Y-H Lee G-S Cheng J Liu PP Feigenbaum L. Gain-of-function Pyrin mutations induce NLRP3 protein-independent interleukin-1beta activation and severe autoinflammation in mice. Immunity. (2011) 34:755–68. 10.1016/j.immuni.2011.02.020 21600797 PMC3129608

[10] Omenetti A Carta S Delfino L Martini A Gattorno M Rubartelli A. Increased NLRP3-dependent interleukin 1beta secretion in patients with familial Mediterranean fever: correlation with MEFV genotype. Ann Rheum Dis. (2014) 73:462–9. 10.1136/annrheumdis-2012-202774 23505242

[11] Gohar F Orak B Kallinich T Jeske M Lieber M von Bernuth H. Correlation of secretory activity of neutrophils with genotype in patients with familial mediterranean fever. Arthritis Rheumatol. (2016) 68:3010–22. 10.1002/art.39784 27333294

[12] Skendros P Chrysanthopoulou A Rousset F Kambas K Arampatzioglou A Mitsios A. Regulated in development and DNA damage responses 1 (REDD1) links stress with IL-1beta-mediated familial Mediterranean fever attack through autophagy-driven neutrophil extracellular traps. J Allergy Clin Immunol. (2017) 140:1378–87.e1313. 10.1016/j.jaci.2017.02.021 28342915

[13] Apostolidou E Skendros P Kambas K Mitroulis I Konstantinidis T Chrysanthopoulou A. Neutrophil extracellular traps regulate IL-1beta-mediated inflammation in familial Mediterranean fever. Ann Rheum Dis. (2016) 75:269–77. 10.1136/annrheumdis-2014-205958 25261578

[14] Lachman HJ Lowe P Felix SD Rordorf C Leslie K Madhoo S. *In vivo* regulation of interleukin 1beta in patients with cryopyrin-associated periodic syndromes. J Exp Med. (2009) 206:1029–36. 10.1084/jem.20082481 19364880 PMC2715040

[15] Jamilloux Y Lefeuvre L Magnotti F Martin A Benezech S Allatif O. Familial Mediterranean fever mutations are hypermorphic mutations that specifically decrease the activation threshold of the Pyrin inflammasome. Rheumatology. (2018) 57:100–11. 10.1093/rheumatology/kex373 29040788

[16] Mende R Vincent FB Kandane-Rathnayake R Koelmeyer R Lin E Hoi AY. Analysis of Serum Interleukin (IL)-1beta and IL-18 in systemic lupus erythematosus. Front Immunol. (2018) 9:1250. 10.3389/fimmu.2018.01250 29930551 PMC5999794

[17] Harvey RF Bradshaw JM. A simple index of Crohn's-disease activity. Lancet. (1980) 1:514. 10.1016/S0140-6736(80)92767-1 6102236

[18] Bilton D Canny G Conway S Dumcius S Hjelte L Proesmans M. Pulmonary exacerbation: towards a definition for use in clinical trials. report from the EuroCareCF working group on outcome parameters in clinical trials. J Cyst Fibros. (2011) 10(Suppl. 2):S79–81. 10.1016/S1569-1993(11)60012-X 21658647

[19] Brown KL Lubieniecka JM Armaroli G Kessel K Gibson KM Graham J. S100A12 serum levels and PMN counts are elevated in childhood systemic vasculitides especially involving proteinase 3 specific anti-neutrophil cytoplasmic antibodies. Front Pediatr. (2018) 6:341. 10.3389/fped.2018.00341 30533405 PMC6266798

[20] Kessel C Lippitz K Weinhage T Hinze C Wittkowski H Holzinger D. Proinflammatory cytokine environments can drive Interleukin-17 overexpression by gamma/delta T cells in systemic juvenile idiopathic arthritis. Arthritis Rheumatol. (2017) 69:1480–94. 10.1002/art.40099 28296284

[21] Lieber M Kallinich T Lohse P Klotsche J Holzinger D Foell D. Increased serum concentrations of neutrophil-derived protein S100A12 in heterozygous carriers of MEFV mutations. Clin Exp Rheumatol. (2015) 33:S113–6. 26486615

[22] Mitroulis I Alexaki VL Kourtzelis I Ziogas A Hajishengallis G Chavakis T. Leukocyte integrins: role in leukocyte recruitment and as therapeutic targets in inflammatory disease. Pharmacol Ther. (2015) 147:123–35. 10.1016/j.pharmthera.2014.11.008 25448040 PMC4324083

[23] Dinarello CA. Overview of the IL-1 family in innate inflammation and acquired immunity. Immunol Rev. (2018) 281:8–27. 10.1111/imr.12621 29247995 PMC5756628

[24] Viganò E Diamond CE Spreafico R Balachander A Sobota RM Mortellaro A. Human caspase-4 and caspase-5 regulate the one-step non-canonical inflammasome activation in monocytes. Nat Commun. (2015) 6:8761. 10.1038/ncomms9761 26508369 PMC4640152

[25] Gaidt MM Ebert TS Chauhan D Schmidt T Schmid-Burgk JL Rapino F Human monocytes engage an alternative inflammasome pathway. Immunity. (2016) 44:833–46. 10.1016/j.immuni.2016.01.012 27037191

[26] De Benedetti F Gattorno M Anton J Ben-Chetrit E Frenkel J Hoffman HM. Canakinumab for the treatment of autoinflammatory recurrent fever syndromes. N Engl J Med. (2018) 378:1908–19. 10.1056/NEJMoa1706314 29768139

[27] Ben-Zvi I Kukuy O Giat E Pras E Feld O Kivity S. Anakinra for colchicine-resistant familial mediterranean fever: a randomized, double-blind, placebo-controlled trial. Arthritis Rheumatol. (2017) 69:854–62. 10.1002/art.39995 27860460

[28] Rubartelli A Cozzolino F Talio M Sitia R. A novel secretory pathway for interleukin-1 beta, a protein lacking a signal sequence. EMBO J. (1990) 9:1503–10. 10.1002/j.1460-2075.1990.tb08268.x 2328723 PMC551842

[29] Keller M Ruegg A Werner S Beer HD. Active caspase-1 is a regulator of unconventional protein secretion. Cell. (2008) 132:818–31. 10.1016/j.cell.2007.12.040 18329368

[30] Monteleone M Stow JL Schroder K. Mechanisms of unconventional secretion of IL-1 family cytokines. Cytokine. (2015) 74:213–8. 10.1016/j.cyto.2015.03.022 25922276

[31] Schroder K Tschopp J. The inflammasomes. Cell. (2010) 140:821–32. 10.1016/j.cell.2010.01.040 20303873

[32] Russo HM Rathkey J Boyd-Tressler A Katsnelson MA Abbott DW Dubyak GR. Active Caspase-1 induces plasma membrane pores that precede pyroptotic lysis and are blocked by lanthanides. J Immunol. (2016) 197:1353–67. 10.4049/jimmunol.1600699 27385778 PMC4976007

[33] Kanneganti A Subbarao Malireddi RK Saavedra PHV Vande Walle L Van Gorp H Kambara H. GSDMD is critical for autoinflammatory pathology in a mouse model of Familial Mediterranean fever. J Exp Med. (2018) 215:1519–29. 10.1084/jem.20172060 29793924 PMC5987922

[34] Magnotti F Lefeuvre L Benezech S Malsot T Waeckel L Martin A. Pyrin dephosphorylation is sufficient to trigger inflammasome activation in familial Mediterranean fever patients. EMBO Mol Med. (2019) 11:e10547. 10.15252/emmm.201910547 31589380 PMC6835204

[35] Chen KW Groß CJ Sotomayor FV Stacey KJ Tschopp J Sweet MJ. The neutrophil NLRC4 inflammasome selectively promotes IL-1beta maturation without pyroptosis during acute Salmonella challenge. Cell Rep. (2014) 8:570–82. 10.1016/j.celrep.2014.06.028 25043180

[36] Heilig R Dick MS Sborgi L Meunier E Hiller S Broz P. The Gasdermin-D pore acts as a conduit for IL-1beta secretion in mice. Eur J Immunol. (2018) 48:584–92. 10.1002/eji.201747404 29274245

[37] Futosi K Fodor S Mocsai A Neutrophil cell surface receptors and their intracellular signal transduction pathways. Int Immunopharmacol. (2013) 17:638–50. 10.1016/j.intimp.2013.06.034 23994464 PMC3827506

[38] Cowland JB Borregaard N. Granulopoiesis and granules of human neutrophils. Immunol Rev. (2016) 273:11–28. 10.1111/imr.12440 27558325

[39] Mishra HK Ma J Walcheck B. Ectodomain shedding by ADAM17: Its role in neutrophil recruitment and the impairment of this process during sepsis. Front Cell Infect Microbiol. (2017) 7:138. 10.3389/fcimb.2017.00138 28487846 PMC5403810

[40] Killock DJ Ivetic A. The cytoplasmic domains of TNFalpha-converting enzyme (TACE/ADAM17) and L-selectin are regulated differently by p38 MAPK and PKC to promote ectodomain shedding. Biochem J. (2010) 428:293–304. 10.1042/BJ20091611 20331435

[41] Walcheck B Kahn J Fisher JM Wang BB Fisk RS Payan DG. Neutrophil rolling altered by inhibition of L-selectin shedding *in vitro*. Nature. (1996) 380:720–3. 10.1038/380720a0 8614468

[42] Lambrecht BN Vanderkerken M Hammad H. The emerging role of ADAM metalloproteinases in immunity. Nat Rev Immunol. (2018) 18:745–58. 10.1038/s41577-018-0068-5 30242265

[43] Molad Y Fridenberg A Bloch K Langevitz P Mukamel M Sulkes J. Neutrophil adhesion molecule expression in familial Mediterranean fever: discordance between the intravascular regulation of beta2 integrin and L-selectin expression in acute attack. J Investig Med. (2004) 52:58–61. 10.1136/jim-52-01-28 14989371

