# Supplementary material for: Gene–Dose Effect of MEFV Gain-of-Function Mutations Determines ex vivo Neutrophil Activation in Familial Mediterranean Fever
Source: Front Immunol. 2020 Jun 11;11:716. doi: 10.3389/fimmu.2020.00716 (PMC7325897; doi:10.3389/fimmu.2020.00716)
Supplement: Supplementary file 1 [file Presentation_1.PPTX]

## Slide 1
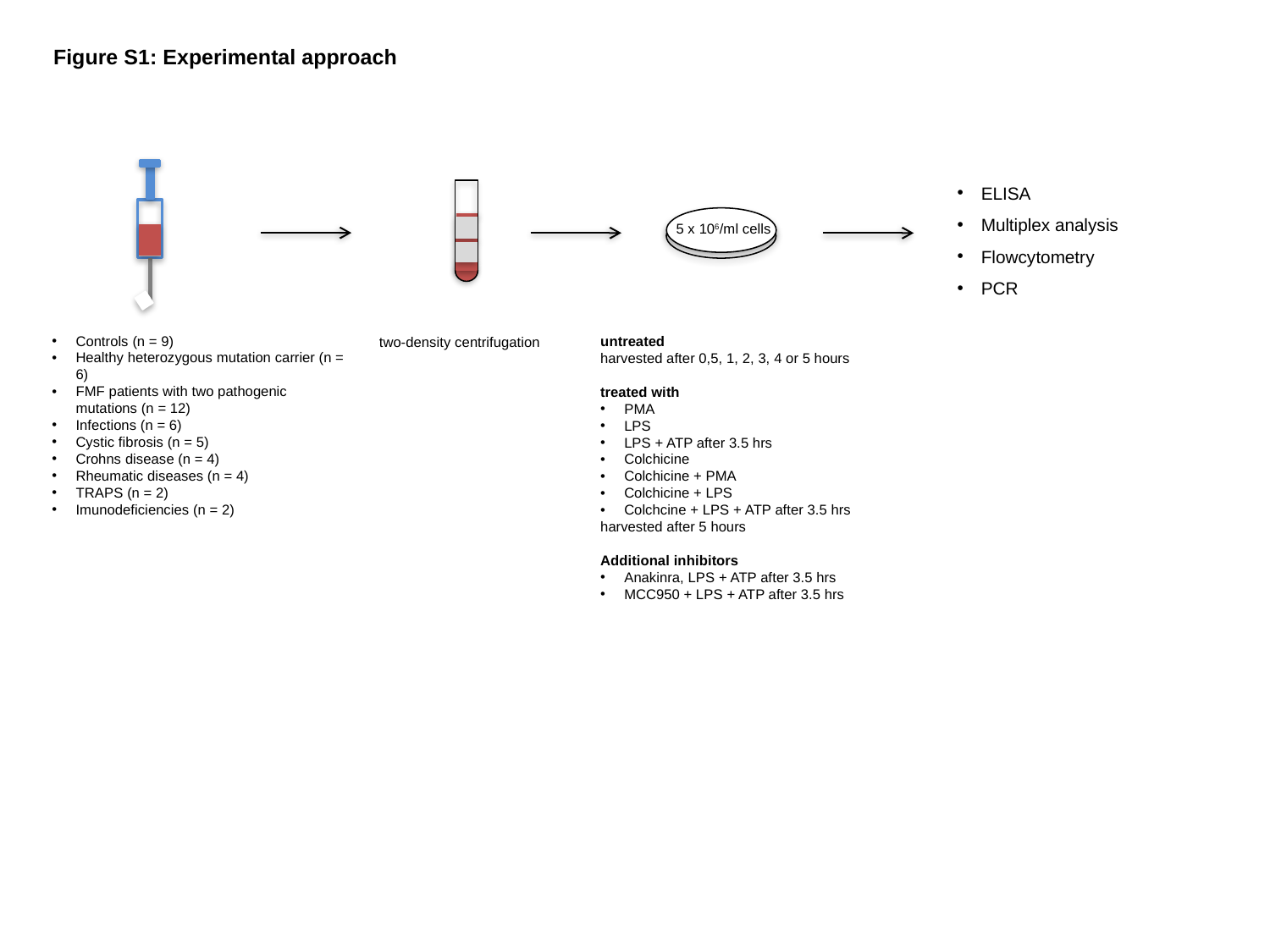

Figure S1: Experimental approach
ELISA
Multiplex analysis
Flowcytometry
PCR
5 x 10^6^/ml cells
Controls (n = 9)
Healthy heterozygous mutation carrier (n = 6)
FMF patients with two pathogenic mutations (n = 12)
Infections (n = 6)
Cystic fibrosis (n = 5)
Crohns disease (n = 4)
Rheumatic diseases (n = 4)
TRAPS (n = 2)
Imunodeficiencies (n = 2)
untreated
harvested after 0,5, 1, 2, 3, 4 or 5 hours
treated with
PMA
LPS
LPS + ATP after 3.5 hrs
Colchicine
Colchicine + PMA
Colchicine + LPS
Colchcine + LPS + ATP after 3.5 hrs
harvested after 5 hours
Additional inhibitors
Anakinra, LPS + ATP after 3.5 hrs
MCC950 + LPS + ATP after 3.5 hrs
two-density centrifugation

## Slide 2
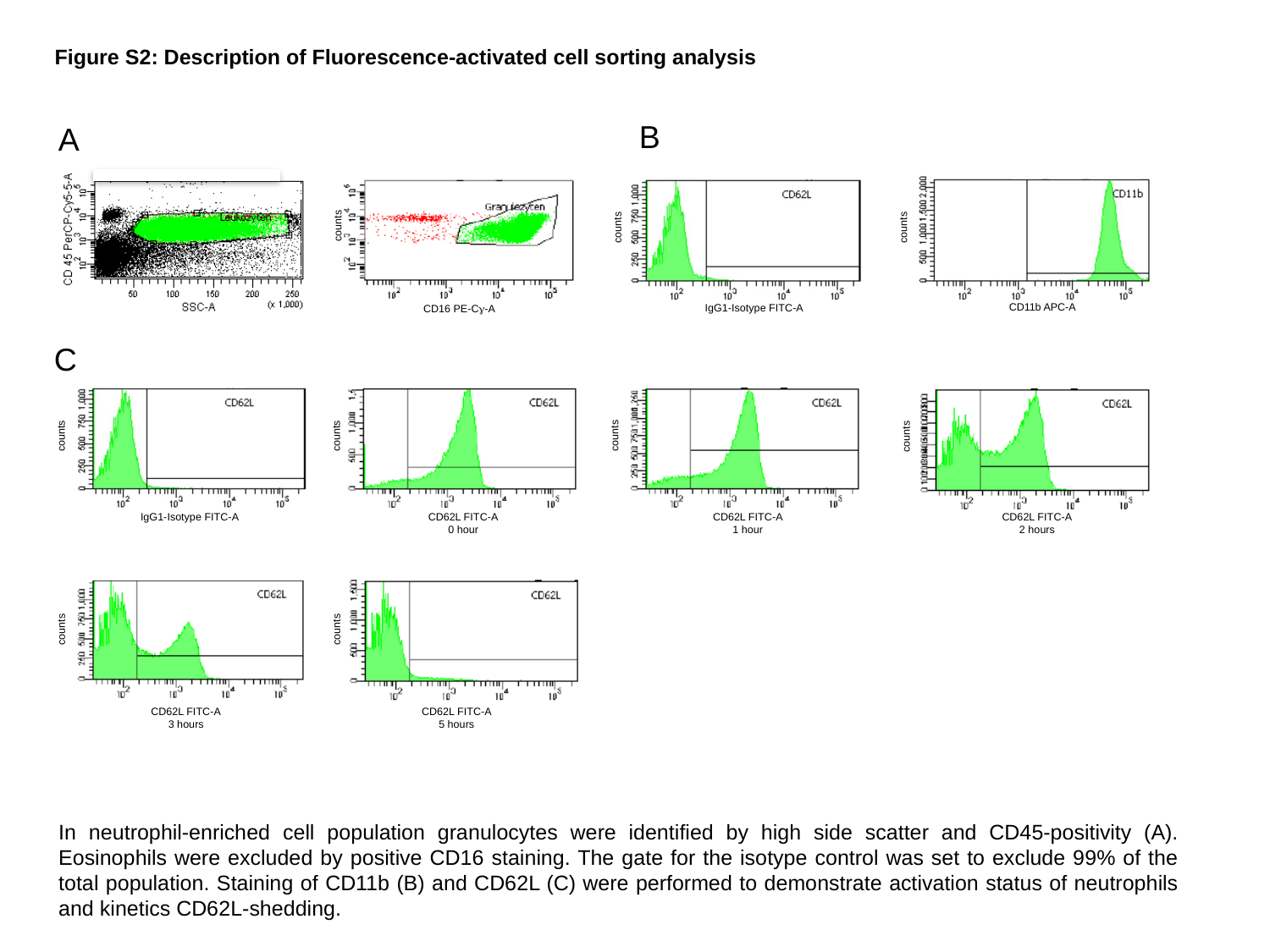

Figure S2: Description of Fluorescence-activated cell sorting analysis
B
A
counts
counts
counts
CD11b APC-A
IgG1-Isotype FITC-A
CD16 PE-Cg-A
C
counts
counts
counts
counts
IgG1-Isotype FITC-A
CD62L FITC-A
0 hour
CD62L FITC-A
1 hour
CD62L FITC-A
2 hours
counts
counts
CD62L FITC-A
3 hours
CD62L FITC-A
5 hours
In neutrophil-enriched cell population granulocytes were identified by high side scatter and CD45-positivity (A). Eosinophils were excluded by positive CD16 staining. The gate for the isotype control was set to exclude 99% of the total population. Staining of CD11b (B) and CD62L (C) were performed to demonstrate activation status of neutrophils and kinetics CD62L-shedding.

## Slide 3
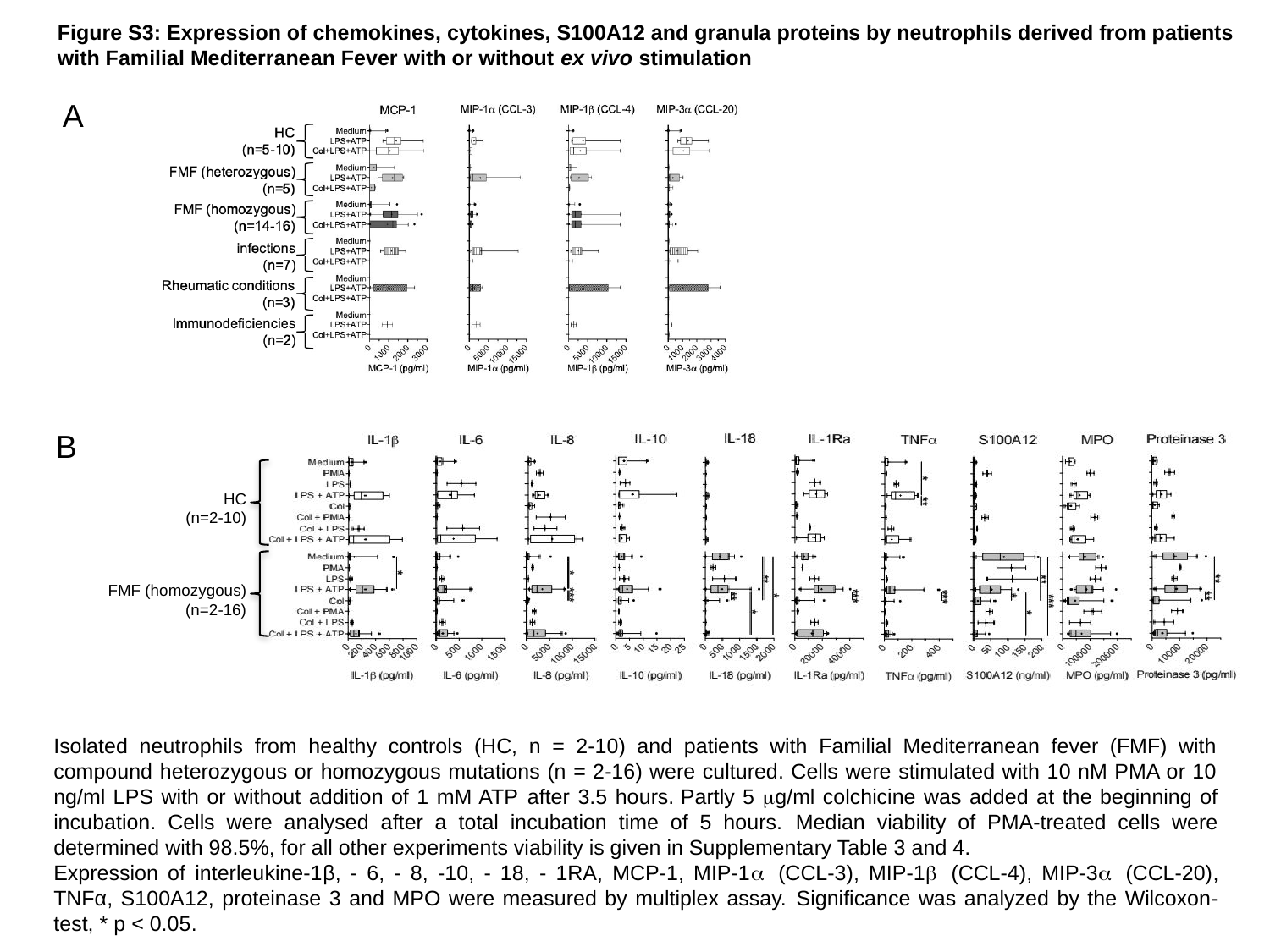

Figure S3: Expression of chemokines, cytokines, S100A12 and granula proteins by neutrophils derived from patients with Familial Mediterranean Fever with or without ex vivo stimulation
A
B
HC
(n=2-10)
FMF (homozygous)
(n=2-16)
Isolated neutrophils from healthy controls (HC, n = 2-10) and patients with Familial Mediterranean fever (FMF) with compound heterozygous or homozygous mutations (n = 2-16) were cultured. Cells were stimulated with 10 nM PMA or 10 ng/ml LPS with or without addition of 1 mM ATP after 3.5 hours. Partly 5 mg/ml colchicine was added at the beginning of incubation. Cells were analysed after a total incubation time of 5 hours. Median viability of PMA-treated cells were determined with 98.5%, for all other experiments viability is given in Supplementary Table 3 and 4.
Expression of interleukine-1β, - 6, - 8, -10, - 18, - 1RA, MCP-1, MIP-1a (CCL-3), MIP-1b (CCL-4), MIP-3a (CCL-20), TNFα, S100A12, proteinase 3 and MPO were measured by multiplex assay. Significance was analyzed by the Wilcoxon-test, * p < 0.05.

## Slide 4
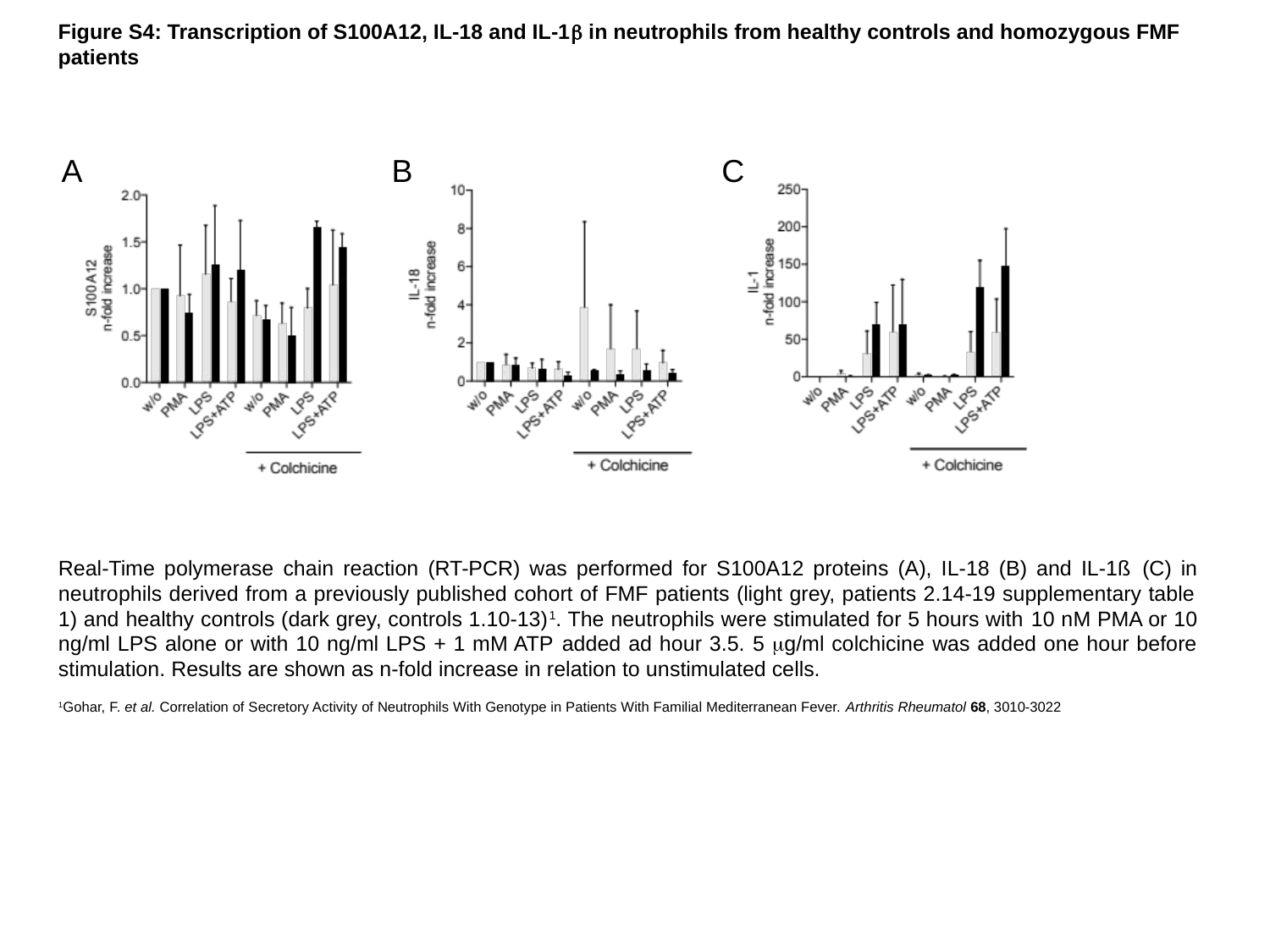

Figure S4: Transcription of S100A12, IL-18 and IL-1b in neutrophils from healthy controls and homozygous FMF patients
A
B
C
Real-Time polymerase chain reaction (RT-PCR) was performed for S100A12 proteins (A), IL-18 (B) and IL-1ß (C) in neutrophils derived from a previously published cohort of FMF patients (light grey, patients 2.14-19 supplementary table 1) and healthy controls (dark grey, controls 1.10-13)1. The neutrophils were stimulated for 5 hours with 10 nM PMA or 10 ng/ml LPS alone or with 10 ng/ml LPS + 1 mM ATP added ad hour 3.5. 5 mg/ml colchicine was added one hour before stimulation. Results are shown as n-fold increase in relation to unstimulated cells.
1Gohar, F. et al. Correlation of Secretory Activity of Neutrophils With Genotype in Patients With Familial Mediterranean Fever. Arthritis Rheumatol 68, 3010-3022

## Slide 5
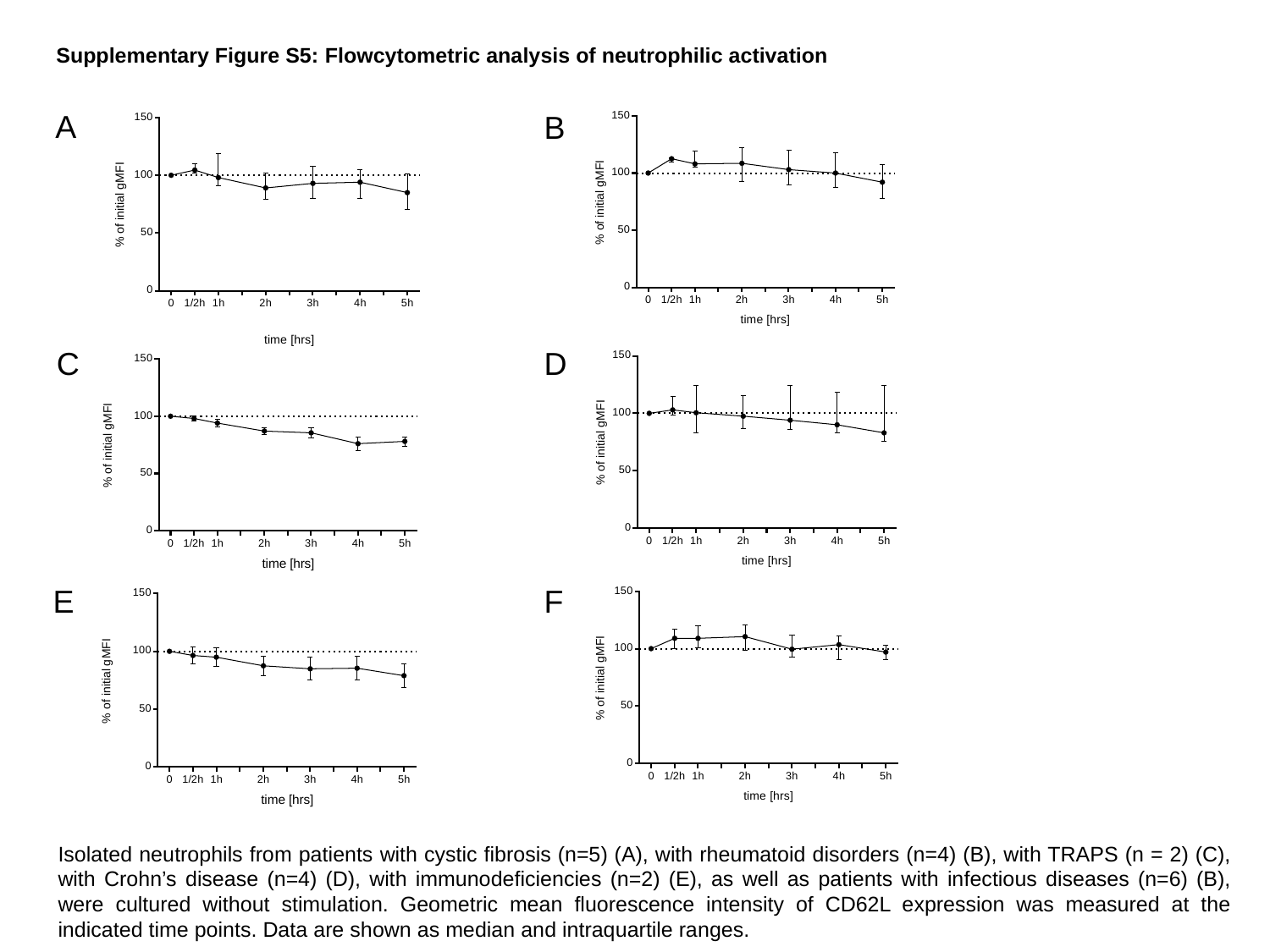

Supplementary Figure S5: Flowcytometric analysis of neutrophilic activation
A
B
D
C
F
E
Isolated neutrophils from patients with cystic fibrosis (n=5) (A), with rheumatoid disorders (n=4) (B), with TRAPS (n = 2) (C), with Crohn’s disease (n=4) (D), with immunodeficiencies (n=2) (E), as well as patients with infectious diseases (n=6) (B), were cultured without stimulation. Geometric mean fluorescence intensity of CD62L expression was measured at the indicated time points. Data are shown as median and intraquartile ranges.

## Slide 6
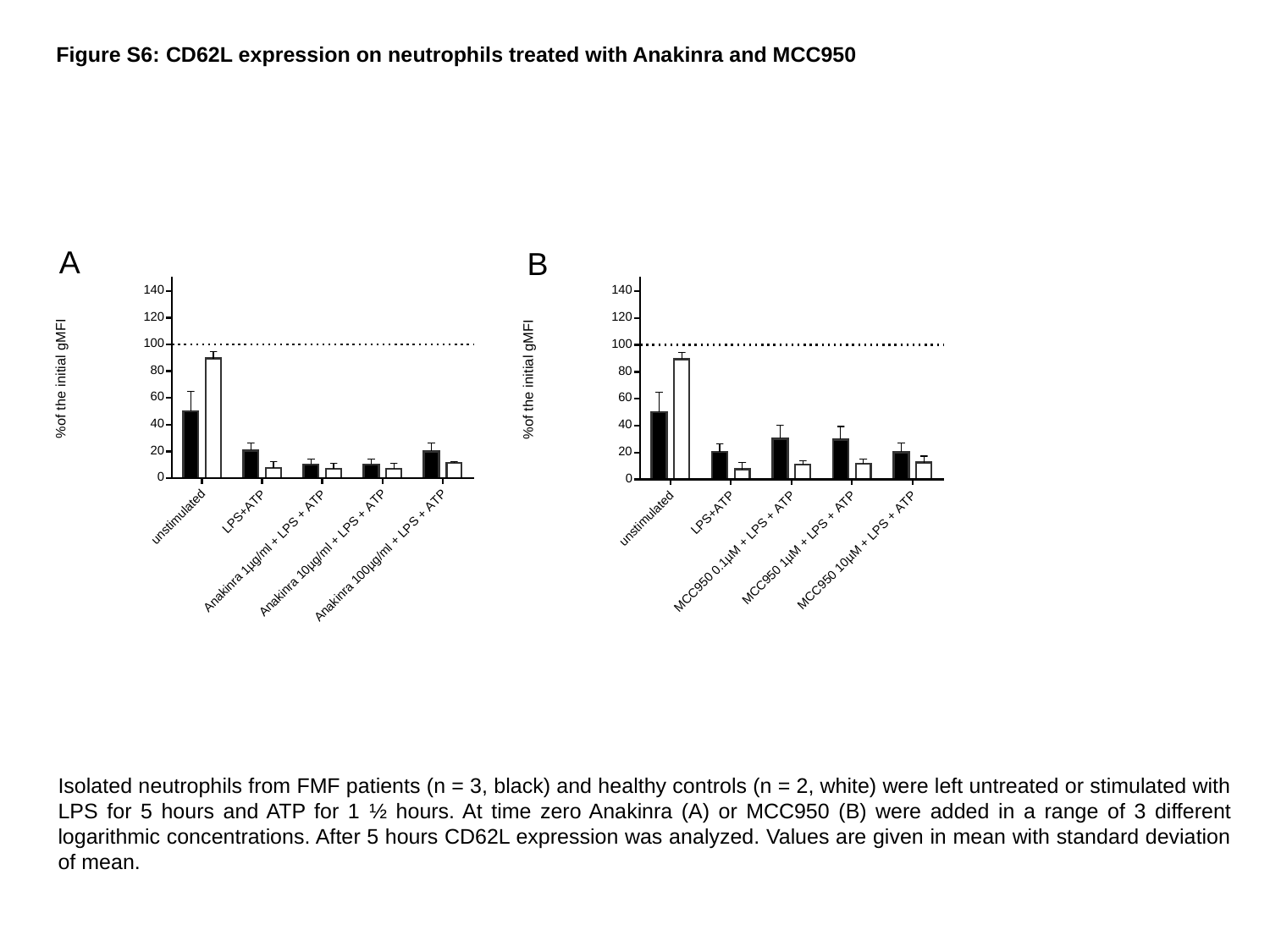

Figure S6: CD62L expression on neutrophils treated with Anakinra and MCC950
A
B
Isolated neutrophils from FMF patients (n = 3, black) and healthy controls (n = 2, white) were left untreated or stimulated with LPS for 5 hours and ATP for 1 ½ hours. At time zero Anakinra (A) or MCC950 (B) were added in a range of 3 different logarithmic concentrations. After 5 hours CD62L expression was analyzed. Values are given in mean with standard deviation of mean.
