# Supplementary material for: Gene–Dose Effect of MEFV Gain-of-Function Mutations Determines ex vivo Neutrophil Activation in Familial Mediterranean Fever
Source: Front Immunol. 2020 Jun 11;11:716. doi: 10.3389/fimmu.2020.00716 (PMC7325897; doi:10.3389/fimmu.2020.00716)
Supplement: Supplementary file 2 [file Data_Sheet_1.docx]

**Supplementary Tables and Methods**

**Table S1: Characteristics of controls and patients**

|  | **age [years]** | **sex [m/f]** | **CRP [mg/l]** | **genotype** | **site/subtype** | **immunmodulatory medication** | **current symptoms** | **severity** |
| --- | --- | --- | --- | --- | --- | --- | --- | --- |
| **1 controls** |  |  |  | **n.a.** | **n.a.** | **n.a.** |  | **n.a.** |
| 1.1 | 30 | m | 1,7 |  |  |  | none |  |
| 1.2 | 27 | f | 1,9 |  |  |  | none |  |
| 1.3 | 55 | f | 6 |  |  |  | none |  |
| 1.4 | 61 | f | 0,6 |  |  |  | none |  |
| 1.5 | 34 | f | 0,7 |  |  |  | none |  |
| 1.6 | 47 | m | 0,7 |  |  |  | none |  |
| 1.7 | 43 | m | < 0,3 |  |  |  | none |  |
| 1.8 | 41 | m | 0,4 |  |  |  | none |  |
| 1.9 | 36 | f | 0,9 |  |  |  | none |  |
| 1.10 | 31 | f | 3,4 |  |  |  | none |  |
| 1.11 | 44 | m | 1,7 |  |  |  | none |  |
| 1.12 | 48 | m | 4,7 |  |  |  | none |  |
| 1.13 | 30 | f | 1,71 |  |  |  | none |  |
| **2 asymptomatic heteroyzgous M694V carriers** |  |  |  |  | **n.a..** | **n.a.** |  | **n.a.** |
| 2.1 | 41 | m | 4,5 | M694V |  |  | none |  |
| 2.2 | 40 | f | 2,3 | M694V |  |  | none |  |
| 2.3 | 51 | f | 5,8 | M694V |  |  | none |  |
| 2.4 | 50 | m | 2 | M694V |  |  | none |  |
| 2.5 | 40 | f | 0,8 | M694V |  |  | none |  |
| 2.6 | 50 | m | 5 | M694V |  |  | none |  |
| **3 FMF** |  |  |  |  | **n.a.** |  |  | **attacks in last year** |
| 3.1 | 25 | m | 112,5  7,6 | M694V/M694V |  | 2,0 mg/d colchicine | none | n.a. |
| 3.2 | 19 | m | 13,6 | M694V/M694V |  | 1,5 mg/d colchicine | none | 12 |
| 3.3 | 15 | f | 44,3 | M694V/M694V |  | 1,5 mg/d colchicine | none | 0 |
| 3.4 | 16 | m | 1,3 | M694V/M694V |  | 1,5 mg/d colchicine | none | 0 |
| 3.5 | 21 | m | 51,7 | M694V/M694V |  | 1.0 mg/d colchicine | none | 0 |
| 3.6 | 21 | f | 0,3 | M680I/M680I |  | 1,5 mg/d colchicine | none | 0 |
| 3.7 | 21 | f | 2,8 | M694V/V726A |  | 1,5 mg/d colchicine | none | 0 |
| 3.8 | 19 | m | 13,6 | M694V/M680I |  | 1.0 mg/d colchicine | none | 0 |
| 3.9 | 15 | m | 5,3 | M694V/V726A/E148Q |  | 1.0 mg/d colchicine | none | 0 |
| 3.10 | 29 | f | 2,3 | M694V/M680I |  | 1,5 mg/d colchicine | none | n.a. |
| 3.11 | 17 | m | 3,8 | M694V/A744S |  | 1.0 mg/d colchicine | none | 0 |
| 3.12 | 18 | m | 4,5 | M694V/V726A |  | 1,5 mg/d colchicine | none | n.a. |
| 3.13 | 24 | m | 81,3 | M694V |  | 1.0 mg/d colchicine | none | 12 |
| 3.14 | 24 | f | 0,7 | M694V/M694V |  | 2,5 mg/d colchicine | none | 5 |
| 3.15 | 22 | m | 6 | E230K/M694V |  | 2,0 mg/d colchicine | none | 1 |
| 3.16 | 26 | m | 7 | M694V/M694V |  | 2,5 mg/d colchicine | none | 10-15 |
| 3.17 | 34 | f | 9 | M694V/M694V |  | 1,5 mg/d colchicine | none | 4-5 |
| 3.18 | 27 | f | 7,1 | M694V/M694V |  | 1,5 mg/d colchicine | none | 2-3 |
| 3.19 | 21 | m | 8,7 | M694V/M694V |  | 2,0 mg/d colchicine | none | 3-4 |
| **4 infections** |  |  |  | **n.a.** | **site** |  |  | **n.a.** |
| 4.1 | 82 | m | 103,1 |  | lung | prednisolon 5 mg  tazobactam/piperacillin | pneumonia |  |
| 4.2 | 52 | m | 73,2 |  | lung | abacavir hemisulfate  lamivudine  doletugravir | pneumonia (HIV (38 cop/ml)) |  |
| 4.3 | 60 | m | 78,1 |  | blood stream | ceftriaxone | sepsis (Staphylococcus haemolyticus) |  |
| 4.4 | 37 | m | 182,8 |  | pericard, pleura | colchicine 0,5mg | pericarditis, pleuritis, suspected bacterial infection |  |
| 4.5 | 64 | m | 91,6 |  | lung | prednisolon 40mg  ampicillin/sulbactam | pneumonia |  |
| 4.6 | 59 | m | 39,6 |  | lung | ampicillin/sulbactam | pneumonia |  |
| **5 cystic fibrosis** |  |  |  |  | **site** |  |  | **Bilton score** |
| 5.1 | 29 | m | 8,6 | F508del/1771-1G>A | lung | cefuroxime  itraconazole | pulmonary exacerbation | 4 |
| 5.2 | 47 | m | 18,4 | F508del/F508del | lung | meropenem  tobramycin | pulmonary exacerbation | 4 |
| 5.3 | 19 | m | 66,1 | F508del/F508del | lung | aztreonam | pulmonary exacerbation | 3 |
| 5.4 | 36 | m | 8,2 | F508del/F508del | lung | ipratropium bromide/fenoterol  azithromycin | pulmonary exacerbation | 4 |
| 5.5 | 29 | f | 35,6 | F508del/F508del | lung | fosfomycin  vancomycin | pulmonary exacerbation | 5 |
| **6 Crohn´s disease** |  |  |  | **n.a.** | **site** |  |  | **Harvey-Bradshaw index** |
| 6.1 | 19 | m | 2,9 |  | gut | adalimumab  azathioprine | diarrhoea | 5 |
| 6.2 | 16 | m | 22,5 |  | gut | adalimumab | diarrhoea | 6 |
| 6.3 | 17 | m | 7,2 |  | gut | Iron sucrose before basis therapy | diarrhoea, anemia | 8 |
| 6.4 | 41 | m | 55,5 |  | gut | vedolizumab | diarrhoea | 8 |
| **7 rheumatic diseases** |  |  |  | **n.a.** | **subtype** |  |  | **DAS28** |
| 7.1 | 74 | m | 3 |  | RA | risedronate sodium  methotrexate  leflunomide | Lumbar radicular syndrom (pain + weakness) | 3.58 |
| 7.2 | 58 | f | 12,1 |  | RA | canakinumab | fatigue, arthritis | 4.45 |
| 7.3 | 19 | f | 6,8 |  | RA | certolizumab  prednisolon 5 mg | arthritis | 4,4 |
| 7.4 | 63 | m | 50,7 |  | adult onset Still´s disease | ursodesoxycholic acid before start of therapy | fever, arthritis, erythema | 4.75 |
| **8 TRAPS** |  |  |  |  | **n.a.** |  |  | **n.a.** |
| 8.1 | 41 | m | 1,9 | T50M |  | canakinumab | none |  |
| 8.2 | 10 | f | 0,3 | T50M |  | canakinumab | none |  |
| **9 immunodeficiencies** |  |  |  |  | **n.a.** |  |  | **n.a.** |
| 9.1 | 24 | m | 8,9 | gp91phox | septic granulomatosis | cotrimoxazole  itraconazole | none |  |
| 9.2 | 53 | m | 1,5 |  | CVID | Immunglobuline substitution  ceftazidime p.i.  latanoprost | Glaucoma  respiratory infections |  |

# CRP – C-reactive protein, RA – rheumatoid arthritis, sJIA – systemic juvenile idiopathic arthritis, TRAPS - tumour necrosis factor receptor-associated periodic syndrome, CVID – chronic variable immunodeficiency, n.a. not applicable. Severity was determined by the Bilton Score (CF), the Harvey-Bradshaw index (Crohn´s disease) and the DAS28 (rheumatic diseases). Control subjects 1.10-13 and FMF patient 3.14-19 have been described previously (light grey lining)^1^.

**Table S2**

**Cell distribution and viability after separation process**

|  | n | neutrophils  [mean / SD] | lymphocytes  [mean / SD] | monocytes  [mean / SD] | eosinophils  [mean / SD] | viability after isolation |
| --- | --- | --- | --- | --- | --- | --- |
| FMF | 12 | 93.2 / 7.1 | 4.7 / 6.4 | 0.2 / 0.1 | 1.8 / 1.0 | 96.9 / 3.4 |
| heterozygous | 6 | 89.0 / 6.9 | 7.0 / 6.3 | 0.8 / 0.6 | 2.9 / 1.7 | 98.8 / 0.4 |
| controls | 6 | 92.4 / 5.4 | 4.0 / 3.4 | 0.4 / 0.3 | 3.1 / 2.4 | 99.0 / 0.0 |
| cystic fibrosis | 6 | 91.9 / 3.9 | 3.8 / 2.2 | 0.7 / 0.6 | 3.5 / 1.7 | 98.3 / 0.8 |
| rheumatic diseases | 4 | 93.2 / 4.2 | 3.7 / 3.1 | 0.8 / 1.0 | 2.1 / 0.9 | 98.5 / 1.0 |
| TRAPS | 2 | 82.4 / 16.4 | 13.1 / 13.0 | 0.4 / 0.1 | 3.8 / 3.2 | 99.0 / 1.4 |
| Crohn´s disease | 4 | 91 / 10.8 | 5.2 / 6.4 | 2.2 / 4.3 | 1.6 / 4.4 | 98.0 / 0.0 |
| infections | 6 | 91.2 / 8.3 | 2.7 / 3.1 | 1.8 / 1.8 | 4.1 / 5.8 | 98.2 / 1.8 |
| immunodefiencies | 2 | 92.7 / 1.3 | 3.2 / 0.4 | 0.4 / 0.0 | 3.7 / 1.6 | 98.0 / 0.0 |
| total | 49 | 90.6 / 9.3 | 5.6 / 8.0 | 0.8 / 1.3 | 2.7 / 2.5 | 98.1 / 1.9 |

Proportions of cell population after two-density centrifugation. Values are given in %. Viability was determined by trypan blue staining. Values for mean are given in %.

**Table S3**

**Cell viability determined by trypan blue staining**

| **condition** | | **FMF patients (n = 11)** | | | **healthy heterozygous mutation carrier (n = 6)** | | | **Control (n = 7=** | | |
| --- | --- | --- | --- | --- | --- | --- | --- | --- | --- | --- |
|  |  | mean | SD | n | mean | SD | n | mean | SD | n |
| after seperation | | 97,0 | 3,4 | 11 | 98,8 | 0,4 | 6 | 98,4 | 1,5 | 7 |
| 5 hours | mock | 93,1 | 7,1 | 7 | 99,0 | 0,0 | 2 | 89,6 | 21,0 | 5 |
|  | LPS + ATP | 96,4 | 3,5 | 7 | 99,0 | 0,0 | 2 | 88,0 | 25,2 | 5 |
|  | colchicine | 98,3 | 1,5 | 7 | 99,0 | 0,0 | 2 | 88,2 | 23,6 | 5 |
|  | LPS + ATP + colchicine | 96,6 | 4,5 | 7 | 99,0 | 0,0 | 2 | 99,0 | 0,7 | 5 |

Cell viability after seperation and 5 hour stimulation. Viability was determined by trypan blue staining and subsequent microscopy. Values for mean are given in %. LPS – lipopolysaccharide, ATP – adenosine triphosphate

**Table S4: Cell viability determined by flowcytometry after propidiumiodide- and annexin-staining**

|  | **FMF patients (n =11)** | | **healthy heterozygous mutation carrier (n = 6)** | | **controls (n = 7)** | |
| --- | --- | --- | --- | --- | --- | --- |
|  | mean | SD | mean | SD | mean | SD |
| mock 5 hours | | | | | | |
| necrosis | 0,4 | 0,4 | 0,1 | 0,1 | 0,1 | 0,1 |
| Late apoptosis/necrosis | 4,0 | 4,6 | 0,6 | 0,5 | 0,6 | 0,2 |
| apoptosis | 5,6 | 5,1 | 5,2 | 6,4 | 8,4 | 5,9 |
| vital | 90,0 | 9,6 | 94,1 | 6,8 | 90,9 | 5,9 |
| colchicine 5 hours | | | | | | |
| necrosis | 0,1 | 0,1 | 0,1 | 0,1 | 0,1 | 0,1 |
| Late apoptosis/necrosis | 0,6 | 0,5 | 0,6 | 0,5 | 0,7 | 0,2 |
| apoptosis | 4,3 | 4,8 | 5,0 | 5,3 | 5,8 | 3,3 |
| vital | 95,0 | 5,2 | 94,4 | 5,7 | 93,4 | 3,4 |
| colchicine + LPS + ATP | | | | | | |
| necrosis | 0,3 | 0,7 | 0,6 | 0,9 | 1,3 | 2,8 |
| Late apoptosis/necrosis | 0,6 | 0,5 | 1,0 | 1,4 | 1,1 | 0,5 |
| apoptosis | 4,2 | 4,9 | 4,7 | 6,4 | 6,0 | 4,9 |
| vital | 94,9 | 5,1 | 93,7 | 7,8 | 91,5 | 4,9 |

Proportion of vital cells determined by trypan blue staining after seperation and after 5 hour stimulation. Viability was determined by flowcytometry after propidiumiodide- and annexin-staining. Values for mean are given in %.
